# Editorial: How to improve neuroscience education for the public and for a multi-professional audience in different parts of the globe

**DOI:** 10.3389/fnhum.2022.973893

**Published:** 2022-07-20

**Authors:** Analía Arévalo, Valeria Abusamra, Guilherme Lepski

**Affiliations:** ^1^Department of Experimental Surgery, Medical School, University of São Paulo, São Paulo, Brazil; ^2^National Scientific and Technical Research Council (CIIPME/CONICET), Buenos Aires, Argentina; ^3^Department of Linguistics, University of Buenos Aires, Buenos Aires, Argentina; ^4^Department of Neurosurgery, Eberhard Karls University, Tübingen, Germany

**Keywords:** neuroscience and education, neuroscience education, teachers, educators, neuroscientists, laypeople, laypeople concepts, neuroscience knowledge

Recent years have seen a growing interest in brain and neuroscience-related knowledge, both among laypeople and those working in critical areas such as health and education. The last decades have also seen an explosion in mass-produced information, as well as the advent of the infamous fake news. In several countries, the search for (and almost parallel supply of) neuroscience-related courses has grown exponentially, but the rate at which this has happened almost guarantees that quality does not match quantity. Another area of rapid growth, especially in North America and Europe, has been the industry of brain-based products, mostly pseudoscientific endeavors that target parents, teachers, schools, and even local governments.

Why is this so important? Neuroscience knowledge—but more importantly—the critical thinking and research skills required to search for and comprehend primary information sources, can help individuals make the right decision regarding their own health and wellbeing. For people in the health industry, it can mean offering the right treatment for their patients. For people in journalism and communication, this can mean translating scientific findings to a lay audience in an easy to understand and accurate way. Finally, for people in education, it can mean properly guiding and preparing generations to come, as well as contributing to the proper allocation of resources.

The gap between cognitive neuroscience and learning is still very conspicuous. And one of the consequences of this distance is the appearance and propagation of myths that in many cases have some scientific support.

Studies conducted in several countries converge on the finding that neuroscience-related knowledge is generally poor among people in all fields, including educators, and in some studies in Europe and South America, it was even observed that heightened interest in neuroscience and even exposure to some short introductory courses actually predicts (paradoxically) a greater belief in neuromyths, combined with an inability to judge information as being real or pseudoscientific. It seems that simply adding quick neuroscience courses to education curricula or in other fields may not be enough to remedy the problem.

The solution may lie in a combination of methods, including courses that specifically cover field-related neuromyths and provide skills that go beyond the content taught, as well as regular, consistent training and access to reliable sources of information. More importantly, this effort requires that neuroscience educators communicate effectively with professionals in various disciplines, including psychologists, health professionals, and educators in other fields.

In this Special Topic, we gathered contributions from researchers in eight countries and four continents who presented original experiments, opinion pieces and descriptions of applied programs that all aim to improve neuroscience-related knowledge in their own corner of the world.

In “*What does the general public know (or not) about neuroscience? Effects of age, region and profession in Brazil*,” Arévalo et al. gathered information about neuroscience-related knowledge among laypeople in Brazil living in all five regions and working in several different fields. The results of the survey filled a gap in knowledge about the largest country in South America, as most previous surveys were conducted in the US, Europe, and Spanish-speaking countries in Latin America. The study revealed overall high neuromyth endorsement, especially among respondents from regions with lower income levels and more limited access to education and the internet, as well as older people. Interestingly, people working in the health field did not perform better than those working in the humanities or exact sciences, revealing poor overall training of professionals in areas that would benefit most from such knowledge. The authors question the quality of the myriad neuroscience courses offered online or at institutions and suggest ways of improving such course offerings.

A response to this problem was offered by Ivanova et al., in “*Advancing neurolinguistics in Russia: experience and implications of building experimental research and evidence-based practices*,” who described their establishment of the Center for Language and Brain at HSE University in Moscow, which started as a small group of scientists and in a short amount of time became a center for cutting edge research and several public outreach programs.

Two other studies conducted in Brazil used fNIRS to study learning in younger students as well as online learning efficiency. Barreto et al. analyzed the interaction between preschool students and their teachers as a way of predicting efficient learning (“*A new statistical approach for fNIRS hyperscanning to predict brain activity of preschoolers' using teacher's*”), while Oku and Sato analyzed an online learning environment in order to outline possible methodological improvements to be implemented (“*Predicting student performance using machine learning in fNIRS data*”).

Another study from Portugal and two from the UK reveal that educators may need some help in this process as well. Through a survey of initial teacher training courses and the availability of brain-related books for educators, Rato et al. reveal the urgent need for developing training curricula for future kindergarten and elementary school teachers in Portugal (“*Looking for the brain inside the initial teacher training and outreach books in Portugal*”). In a perspective article (“*The Learning Styles neuromyth is still thriving in medical education*”) and systematic review (“*How common is belief in the Learning Styles neuromyth, and does it matter? A pragmatic systematic review*”), Newton et al. and Newton and Salvi reveal the widespread endorsement of the Learning Styles neuromyth among educators in different areas despite no empirical evidence for it and discuss the implications of this belief on education.

So, what can be done to aide teachers in this process? In “*On neuroeducation: why and how to improve neuroscientific literacy in educational professionals*,” Jolles and Jolles present a proposal that includes four themes of neuroscience content “that every teacher should know.” The authors emphasize the need for interdisciplinary involvement in such efforts. Also, in “*Teaching the science in neuroscience to protect from neuromyths: from courses to fieldwork*,” Carboni et al. describe a set of activities being conducted in Uruguay since 2013 that aim to bridge the gap between Education and Neuroscience and involve activities that bring together educators and scientists to work on research projects, as well as a course that focuses on the applications of Neuroscience to Education. These authors emphasize the need to provide educators with a deeper understanding of the science to make their own better educational decisions. In “*Neuroscience concepts changed teachers' views of pedagogy and students*” (Chang et al.), an educational neuroscience concepts course offered a group of K-12 teachers in Texas a lens to reconsider, re-envision and re-design their lessons. Two other innovative studies were conducted with educators in Liberia: in one, training in neuroscience and mental health development improved teacher self-efficacy, self-responsibility for student outcomes, and motivation to teach (“*Tiered neuroscience and mental health professional development in Liberia improves teacher self-efficacy, self-responsibility, and motivation*,” Brick et al.), and in the other, mental health training improved teachers' understanding of their students' mental and emotional difficulties, reduced their use of verbal and corporal punishment, and helped them establish positive rewards systems (“*Training-of-trainers neuroscience and mental health teacher education in Liberia improves self-reported support for students*,” Brick et al.). And in an effort to improve teaching neuroscience history, Schleim offers a new perspective on the classic case of Phineas Gage [“*Neuroscience education begins with good science: communication about Phineas Gage (1823-1860), one of neurology's most-famous patients, in scientificarticles*”].

Finally, in “*Neuroscience outside the box: from the laboratory to discussing drug abuse at schools*,” Machado do Vale et al. offer perspectives on how scientists can engage educators, students, policymakers, and the public at large to effect real change in society through a neuroscientific perspective.

The aim of this collection of 14 articles was to join forces with a large network of neuroscience and education professionals and inspire and guide others with similar interests toward effective solutions. Forging strong links between domains requires double literacy: teachers need to become “neuroscientifically literate” and neuroscientists have to become “educationally literate.”

We hope these and future work can continue to improve neuroscience education for the public and for a multi-professional audience around the globe.

## Author contributions

All authors listed have made a substantial, direct, and intellectual contribution to the work and approved it for publication.

## Conflict of interest

The authors declare that the research was conducted in the absence of any commercial or financial relationships that could be construed as a potential conflict of interest.

## Publisher's note

All claims expressed in this article are solely those of the authors and do not necessarily represent those of their affiliated organizations, or those of the publisher, the editors and the reviewers. Any product that may be evaluated in this article, or claim that may be made by its manufacturer, is not guaranteed or endorsed by the publisher.

